# PyPanda: a Python package for gene regulatory network reconstruction

**DOI:** 10.1093/bioinformatics/btw422

**Published:** 2016-07-10

**Authors:** David G.P. van IJzendoorn, Kimberly Glass, John Quackenbush, Marieke L. Kuijjer

**Affiliations:** ^1^Department of Pathology, Leiden University Medical Center, 2300RC Leiden, The Netherlands; ^2^Channing Division of Network Medicine, Department of Medicine, Brigham and Women’s Hospital, Harvard Medical School, Boston, MA 02215, USA; ^3^Department of Biostatistics and Computational Biology, Dana-Farber Cancer Institute, Boston, MA 02215, USA; ^4^Department of Biostatistics, Harvard T.H. Chan School of Public Health, Boston, MA 02215, USA; ^5^Department of Cancer Biology, Dana-Farber Cancer Institute, Boston, MA 02215, USA

## Abstract

**Summary:** PANDA (Passing Attributes between Networks for Data Assimilation) is a gene regulatory network inference method that uses message-passing to integrate multiple sources of ‘omics data. PANDA was originally coded in C ++. In this application note we describe PyPanda, the Python version of PANDA. PyPanda runs considerably faster than the C ++ version and includes additional features for network analysis.

**Availability and implementation:** The open source PyPanda Python package is freely available at http://github.com/davidvi/pypanda.

**Contact:** mkuijjer@jimmy.harvard.edu or d.g.p.van_ijzendoorn@lumc.nl

## 1 Introduction

Accurately inferring gene regulatory networks is one of the most important challenges in the analysis of gene expression data. Although many methods have been proposed (Altay *et al.*, 2011; Ernst *et al.*, 2008; Faith *et al.*, 2007; Lemmens *et al.*, 2006), computation time has been a significant limiting factor in their widespread use. PANDA (Passing Attributes between Networks for Data Assimilation) is a gene regulatory network inference method that uses message passing between multiple ‘omics data types to infer the network of interactions most consistent with the underlying data (Glass *et al.*, 2013). PANDA has been applied to understand transcriptional programs in a variety of systems (Glass *et al.*, 2014, 2015b; Lao *et al.*, 2015).

Here we introduce PyPanda, a Python implementation of the PANDA algorithm, following the approach taken in Glass *et al.* (2015a) and optimized for matrix operations using NumPy (van der Walt *et al.*, 2011). This approach enables the use of fast matrix multiplications using the BLAS and LAPACK functions, thereby significantly decreasing run-time for network prediction compared with the original implementation of PANDA, which was coded in C ++ and used for-loops (Glass *et al.*, 2015a). We observe further speed increase over the C ++-code because PyPanda automatically uses multiple processor-cores through the NumPy library. We have also expanded PyPanda to include common downstream analyses of PANDA networks, including the calculation of network in- and out-degrees and the estimation of single-sample networks using the recently developed LIONESS algorithm (Kuijjer *et al.*, 2015).

## 2 Approach

### 2.1 Comparing PANDA C ++-code to Python-code

We compared the C ++-code and Python-code versions of PANDA using several metrics. First, we assessed the two implementations by comparing the number of lines of code. Using the *cloc* utility we counted the number of lines of C ++-code and Python-code. The C ++-code counted 1132 lines of code. The Python-code counted 258 lines of code, significantly shorter (4.4 times) than the C ++-code. The Python-code also includes features such as the LIONESS equation and in- and out-degree calculation. Without these features the Python-code is only 155 lines of code. Because the Python implementation is much more concise than the C ++-code it is easier to interpret and modify.

Next we performed a speed comparison test between the C ++-code and the Python-code. We used built-in timing functions for both languages, directly before and after the message passing part of the code as this is the step that consumes the most time (Glass *et al.*, 2015a). For the C ++-code, we used *gettimeofday()* to record time in milliseconds before and after the message passing algorithm. For the Python code we implemented the *time.time()* function around the message passing algorithm. The C ++-code was compiled using the *clang* compiler (version 3.8.0) with speed optimization flag -O3. Python (version 2.7.10) was used with NumPy (version 1.10.1) using the BLAS and LAPACK algebraic functions. All analyses were run on a server running ×86_64 GNU/Linux.

The speed of the network prediction was tested using simulated networks of *Ne*=* Na* dimensions, where *Ne* is the number of effector nodes and *Na* is the number of affected nodes. For each of several different network sizes (Ne=Na=125 to Ne=Na=2000 nodes, in steps of 125) we generated ten random ‘motif data’ networks according to the method described in Glass *et al.* (2015a). We then ran the Python and C ++ versions of PANDA using these simulated motif data together with identity matrices for the protein-protein interaction and co-expression information. For runs on the same initial ‘motif data’ networks, we verified that the C ++-code and Python-code returned exactly the same output network, as expected due to the deterministic nature of PANDA.

**Fig. 1.**
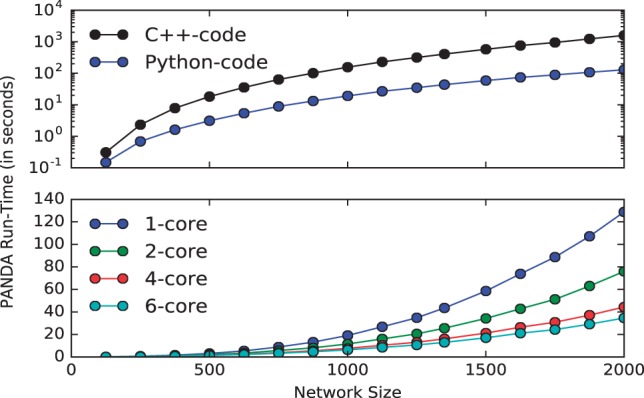
Speed comparison for network reconstruction on networks of different sizes using **(A)** the C ++-code and the Python-code, **(B)** the Python-code running on a single CPU compared with multicore (6 CPU cores)

The C ++-code only uses one CPU core. In comparing the C ++-code with the Python-code using a single core, we found a 2.07-fold speed-up relative to the C ++-code for the smallest network (Ne=Na=125) tested. The speed increase of the Python-code over the C ++-code became larger as the network size increased. For example, the Python-code performed 12.31 times faster for the largest network (Ne=Na=2000) (Fig. 1A). Recorded run times across the ten random networks had a standard deviation of 0.04s and 2.59 s for the smallest (Ne=Na=125) and largest (Ne=Na=2000) networks, respectively using the C ++ code. Using the Python code these were reduced to 0.03s and 0.099 s.

Given the abundance of multicore computing resources currently available, we also tested the speed increase when running the Python-code on multiple cores compared with running the Python-code on a single core. We found that for the smallest network the speed was 1.45 times faster when using 6 cores compared with using only a single core; for the largest network the speed increase was 3.7-fold (Fig. 1B).

This increase in speed enables reconstruction of networks with larger numbers of regulators and target genes. For example, using the Python-code significantly decreases the time required to infer a human gene regulatory network (*Ne* = 1000, *Na* = 20 000), from ∼18 h with the C ++-code to only about 2 h with the Python-code. This speed-up is especially important as transcription factor motif databases are frequently updated to include more motifs. Further, the decreased running time helps to enable the estimation of network significance by making the use of bootstrapping/jackknifing methods much more feasible.

### 2.2 Additional features

In addition to reconstructing one regulatory network based on a data set consisting of multiple samples, PyPanda can also reconstruct single-sample networks using the LIONESS algorithm (Kuijjer *et al.*, 2015). In PyPanda, the LIONESS method uses PANDA to infer an ‘aggregate’ network representing a set of *N* input samples, infers a network for *N* – 1 samples, and then applies a linear equation to estimate the network for the sample that had been removed. The process is then repeated for each sample in the original set, producing *N* single-sample networks. PyPanda can also use LIONESS to reconstruct single-sample networks based on Pearson correlation.

PyPanda also includes functions to calculate in-degrees (the sum of edge weights targeting a specific gene) and out-degrees (the sum of edge weights pointing out from a regulator to its target genes). These summary metrics can be used for downstream network analysis (Glass *et a*l., 2014).

## 3 Conclusion

PANDA is a proven method for gene regulatory network inference but, like most sophisticated network inference methods, its runtime has limited its utility. The Python implementation of PANDA uses matrix operations and incorporates the NumPy libraries, resulting in a significant simplification of the code and a dramatic increase in computing speed, even on a single processor. When applied to a test data set and run on multiple processing cores, this increase in speed was even greater, decreasing processing times by a factor of 46 relative to the original C ++-code. This creates opportunities to greatly expand the use of PANDA and to implement additional measures of network significance based on bootstrapping/jackknifing. PyPanda also includes the LIONESS method, which allows inference of single-sample networks, as well as a number of other useful network metric measures. The open source PyPanda package is freely available at http://github.com/davidvi/pypanda.
